# The complete mitochondrial genome of the *Agrocybe aegerita*, an edible mushroom

**DOI:** 10.1080/23802359.2017.1398618

**Published:** 2017-11-07

**Authors:** Li-Ming Xu, Damien Daniel Hinsinger, Guo-Feng Jiang

**Affiliations:** a Biology Institute, Guangxi Academy of Sciences, Nanning, Guangxi, PR China;; b Biodiversity Genomics Team, Plant Ecophysiology & Evolution Group, Guangxi Key Laboratory of Forest Ecology and Conservation, College of Forestry, Guangxi University, Nanning, Guangxi, PR China;; c State Key Laboratory for Conservation and Utilization of Subtropical Agro-Bioresources, Guangxi University, Nanning, Guangxi, PR China

**Keywords:** Fungi, tea tree mushroom, *Agrocybe aegerita*, mitogenome, evolution

## Abstract

*Agrocybe aegerita* is a medicinally and nutritionally important edible basidiomycete. Despite previous phylogenetic studies, the taxonomy of *A. aegerita* complex remains unclear due to lacking of resolutive data. Herein, the complete mitochondrial genome of *A. aegerita* is reported and analyzed. The mitogenome length was 116,329 bp, with a GC content of 27.6%, include 17 typical protein-coding genes, two ribosomal protein genes (*rps3*), two ribosomal RNA genes and a set of 32 transfer RNA genes. A phylogenetic analyses using complete mitogenome in Agaricales showed that *A. aegerita* is closely related to the genus *Pleurotus* and represents a clade clearly independent from other Agaricales species.

*Agrocybe aegerita* (Basidiomycota, Agaricomycetes, Agaricales, Strophariaceae) (Matheny et al. 2006; Uhart et al. 2007) is used as a nutritious food and herbal medicinal around the world (Wasser and Weis 1999; Zhao et al. 2003; Tsai et al. 2006; Thushara et al. 2008). *Agrocybe aegerita* is cultivated and is a biological model for the developmental genetic engineering (Barroso et al. 1995; Noël and Labarère 1994). *Agrocybe aegerita* has been characterized as a multispecies complex due to a wide range of variation of morphological and physiological characters, the disordered designation has hindered the development of breeding programs (Singer 1986; Uhart et al. 2007; Chen et al. 2012). Using rDNA and ITS sequence in *A. aegerita* complex showed a high diversity in the species/varieties collected from different place (Uhart et al. 2007; Chen et al. 2012). However, the taxonomical status of this species remains unclear, and no complete mitogenome is available to date. Here, we report the complete mitochondrial genome of *A. aegerita* to provide new genetic resources and to uncover the taxonomical status of this species.

The strain SWS_17 of *A. aegerita* is conserved in the Biology Institute, Guangxi Academy of Sciences (Nanning, Guangxi, PR China). Total genomic DNA was extracted from an individual previously grown on Potato Dextrose Agar (PDA) medium at 25 °C for 2 weeks, using the Promega Genomic DNA Purification System (Promega, Madison, WI). Library construction and sequencing were processed by Novogene (Beijing, China), according to the Illumina HiSeqX-ten system manufacturer (Illumina Inc., San Diego, CA) instructions. The complete mitogenome was de novo assembled using org.asm v0.2.05 (available at http://pythonhosted.org/ORG.asm/) followed by manual checking in Geneious R9 v9.1.6 (Biomatters Ltd, Auckland, New Zealand) as described previously (Hinsinger and Strijk 2016; Jiang et al. 2016). Genome annotation was performed with the on line program DOGMA (Wyman et al. 2004) and manually checked in Geneious. The complete mitogenome sequence of *A. aegerita* was submitted to GenBank under accession no. MF979820. Phylogenetic analysis was performed with Phyml 3.1 to construct a maximum-likelihood (ML) tree including 10 mitochondrions available of Agaricales in GenBank (see Figure 1). Nucleotide substitution models were tested by jmodeltest (Posada 2009).

**Figure 1.**
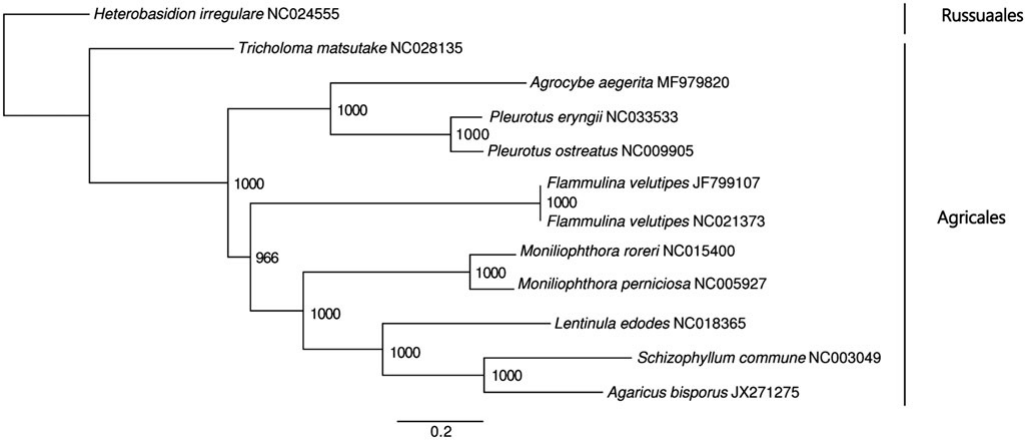
ML phylogenetic tree of the 10 available mitochondrial sequences of Agaricales in GenBank, plus the mitochondrial sequence of *Agrocybe aegerita*. The tree is rooted with *Heterobasidion irregulare*. Bootstraps values (1000 replicates) are shown at the nodes. Scale in substitution per site.

The complete mitogenome of *A. aegerita* was 116,329 bp in length, containing two pairs of large reverse repeat regions (4061 bp and 24,473 bp in length, respectively). The overall GC content was 27.6%, including 17 protein-coding genes, two ribosomal protein gene *rps3*, 32 tRNA genes, and two rRNA genes (*rnl* and *rns*). The protein coding genes involved in respiration and oxidative phosphorylation included the three ATP synthase subunits (*atp6*, *atp8* and *atp9*), the seven NADH dehydrogenase subunits (*nad1*, *nad2*, *nad3*, *nad4*, *nad4L*, *nad5* and *nad6*), the three cytochrome oxidase subunits (*cox1*, *cox2 and cox3*), and the apocytochrome b (*cob*). The 32 tRNA genes ranged in size from 71 bp to 87 bp, and coded for all 20 standard amino acids. The ML tree showed that *A. aegerita* is sister to the genus *Pleurotus* forming a separated clade from other Agaricales species (Figure 1). Support was high for all nodes, but the close relationship between *Agrocybe* and *Pleurotus* differs from the previous studies (Matheny et al. 2006), suggesting that adding more complete mitogenome will greatly help to retrieve a high resolution and highly supported phylogeny. With this first complete mitochondrial genome of *Agrocybe aegerita*, we expect identification in *Agrocybe* will be facilitated, and breeding programs improved and expanded.
